# Evaluation of therapeutic relationship skills training for mental health professionals: the Therapeutic Relationship Enabling Programme (TREP)

**DOI:** 10.15694/mep.2019.000112.1

**Published:** 2019-05-28

**Authors:** Faith Liao, David Murphy

**Affiliations:** 1 University of Nottingham

**Keywords:** Communication skills, Competence Aqucisiton, Therapeutic Relationship, Medical Education, Education in Psychiatry, Curriculum Development/Evaluation, Person-Centred Experiential Approach, Quasi-Experiment, New technology

## Abstract

This article was migrated. The article was marked as recommended.

**Background:** A 3-day workshop in Taiwan, developed in accordance with Carl Rogers’ person-centred theory, used an experiential-learning pedagogy and a helping learning technology mPath. This study aimed to evaluate the effectiveness of a short-term course for mental health professional students assessing to the acquisition of therapeutic relationship competencies.

**Objective:**To evaluate the training effects and investigate any changes in the level of therapeutic relationship competence of the participants before, at the end and two weeks after the intervention.

**Methods:**A sample of 59 mental health professional students from 7 medical schools studying in nursing, occupational therapy, medicine, clinical psychology and other specialities with the completion of psychiatry-relevant courses. 26 of 59 mental health professional students volunteered to form the experimental group, and the controls were recruited using the snowball sampling technique. All of them completed the Barrett-Lennard Relationship Inventory OS-40 three times. Mean values and statistical significance tests were computed to compare the results.

**Results:**Within 3 days, the mental health professional students in the experimental group (N=26) completed the Therapeutic Relationship Enabling Programme (TREP) and showed a statistically significant level of change (Mean Difference= +9.5, *p=* 0.002), which was in contrast to the outcome of the control group (N=33, Mean Difference= +0.18, *p=* 0.683), in the therapeutic relationship competences. The effecting growth curve of therapeutic relationship competence in the experimental group continually inclined two weeks after the intervention (Mean Difference= +19.423, *p=* 0.000) while the control group reflected a decline in therapeutic relationship competence (Mean Difference= -0.515, *p=* 0.812).

**Conclusions:** A person-centred-theory-based training workshop with the use of a specially designed technology enhanced Taiwanese mental health professional students’ learning on therapeutic relationship competences. A further investigation into learning person-centred therapeutic relationship qualities in the workshop as an innovative pedagogy and learning approach for medical education would be recommended.

## Introduction

Physicians are trained for the major purpose of preparing themselves to provide a fusion of medical science and social science in which emphasis is often placed upon the importance of developing empathy towards patients in medical practice. This is specifically the case when their task is to learn about the psychiatric context within their medical education. As literature from the American Association of Medical Colleges (AAMC), General Medical Council (GMC) in the United Kingdom and the Liaison Committee on Medical Education (LCME) in Canada and the United States shows, empathy enhancement is one of the essential goals for learning in medical education. This is to ensure medical students’ competency in developing and maintaining therapeutic relationships which can be relevant to patients’ prognosis ( Stephien and Baernstein, 2006; Dale, Bhavsar and Bhugra, 2007; Shaprio, 2008; Bayne, 2011).

Psychiatry is an important branch of medicine. As such, medical trainings have seen increases to the total number of teaching hours assigned to psychiatry rotations, revising and expanding psychiatric teaching programs throughout the whole course, developing clinical placements in hospital wards starting in connection with other medical services, and developing the technique of psychiatric examination to parallel that of the physical diagnosis process ( Dale, Bhavsar and Bhugra, 2007; Bayne, 2011; Vestermark, 1952; Thompson, 1953; Law *et al.,* 2011). This study examines the effectiveness of a therapeutic relationship training programme (TREP) for medical and psychiatry-related health professionals that used a combination of experiential learning methods supported by new technologies.

One of the most important elements of all psychiatric treatments is the relationship between the healthcare professional and the patient. Qualitative researchers have suggested that the therapeutic relationship in mental health care plays an essential role in patients’ recovery from severe mental illness, such as schizophrenia ( McCabe and Priebe, 2004)and that the relationship directly affects outcomes ( Bendapudi *et al.,* 2006)and this has been shown in studies that have investigated self or peer-rating assessments, observation studies, quasi-experiments, and interviews ( Priebe *et al.,* 2011; Ditton-Phare *et al.,* 2017). Conducting multi-faceted studies in therapeutic relationships and providing effective psychiatric healthcare are suggested to be increasingly crucial in response to the rise in psychological distress in the population ( Ditton-Phare *et al.,* 2017; Fu *et al.,* 2013; World Health Organization, 2014; Pescosolido *et al.,* 2013). However, due to the heterogeneity of studies there is still little known about the therapeutic relationship within different psychiatric approaches ( Ditton-Phare *et al.,* 2017; Maguire and Pitceathly, 2002), and the frameworks of the therapeutic relationship in psychiatric healthcare remain unclear ( Bayne, *2011*; Priebe *et al.,* 2011). Thus, developing mental health professionals’ therapeutic relationship knowledge and skills could be one of the essential focuses for the future of psychiatric and psychiatry-related professional education ( Alberts and Eldstein, 1990; Messina, Sambin and Palmieri, 2013).

Therapeutic Relationship in Psychiatry

In mainstream mental health care, the definitions and approaches of therapeutic relationship are varied; such as the term *therapeutic relationship ( Alexander and Coffey,1997)* meaning a therapist and a patient that engage with each other to effect beneficial change in the patient, *therapeutic alliance* ( Clarkin, Hurt and Crilly,1987)which means creating a bond between patient and therapist to formulate and apply judgment precisely and help patients define and reach their goals, the *helping relationship* ( Goering and Stylianos,1988)which is a relationship between the helper and the helpee facilitating the quality of the relationship with five characteristics: listening attentively, understanding the other person’s point of view, accepting the person non-judgmentally, caring enough to be committed and involved (but not overly involved), and being genuine, and lastly the *working alliance* ( Gehrs and Goering, 1994) which consists of three parts: tasks, goals and bond agreed by both parties to help reach the client’s goals.

Regardless of the various approaches to the therapeutic relationship, studies have indicated that a mutual experience of the therapeutic relationship is an influential factor to assist the clients through the treatment ( Cornelius-White *et al.,* 2018; McGuire, McCabe and Pierbe, 2001). Moreover, a systematic review of research identified 129 studies between 1990 to 2009 that had addressed the correlation between the patients’ prognosis and the therapeutic relationship. The review suggested there was an overall small effect size *r*= .22 ( Martin, Garsle and Davis, 2000). 33 of the 129 studies were identified as investigations on whether the therapeutic relationship predicted the outcome of the treatment; 22 of these studies used clinician- or patient-rated measures and results reported in 3 of 6 studies with hospitalized patients showed that a better therapeutic relationship was associated with fewer hospitalizations, 3 of 10 measured the level of patient’s symptoms and reported that the therapeutic relationship was one of the most influential factors for symptom reduction, and 6 out of 6 studies evaluating the patient’s functioning showed significant associations with the therapeutic relationship ( Priebe *et al.,* 2011).

A systematic review of current approaches of communication skills for those in the psychiatric and psychiatry-related professions indicated an improvement in empathy and interview skills after experiencing therapeutic-relationship-related training protocols ( Ditton-Phare *et al.,* 2017). Medical students in a quasi-experimental study on learning doctor-patient communication reported that 84 students (male=32, female=52, mean age=21.9) showed significant learning progress on communication skills (p<0.001) at the beginning and the end of a 39-hour course over two semesters. The course involved tutors with groups of 8-12 students working together, using doctor-patient role play, giving and receiving feedback, analysing video, and a video exam ( Cämmerer, Martin and Rockenbauch, 2016). Another quasi-experimental study on psychiatric nurses’ communication skills training was conducted as a short-term course with lectures, problem-solving, brainstorming, members sharing experiences and discussion, and using personal computer and whiteboards as educational tools. The study discovered that the level of stress of the random-assigned members in the experimental group (N=23, mean stress score difference = -.1) decreased significantly one month after the intervention while and the control group’s stress level (N=22, mean stress score difference = +.4) continued to increase. Ghazavis concluded that psychiatric nurses are often influenced by the stressful working environment which leads to desensitizing in therapeutic relationship with their patients, and he also suggested providing an appropriate working environment forward nurses to learn communication skills that could lower the workload and improve the therapeutic relationship with patients ( Ghazavi, Lohrasbi and Mehrabi, 2010).

Despite these recent studies, the underlying premise of the therapy relationship in psychiatric education remains confusing as it can be positioned from multiple theoretical perspectives. There are at least six conceptual and theoretical frameworks of mental health professional training that have traditionally been applied, and five of them have emphasized the importance of developing therapeutic relationship competence during training. For example, the *role theory* focuses upon the functions and patterns of behaviours of the practitioners and patients in the relationship, the *social constructionism* looks at the process of patients’ interpretation about their experience through the communication with the practitioners, the *systems theory* is functioning as a reconstruction of patients’ external systems, such as their family, in the therapeutic settings, *social psychology* emphasizes the interpersonal interaction between the practitioners and patients; and lastly, the *cognitive behaviourism* bridges patients’ cognition and behaviours by facilitating a working relationship in the psychiatric settings ( McGuire, McCabe and Pierbe, 2001).

Carl Rogers, a pioneer of Humanistic Psychology and psychotherapy and specifically the person-centred approach, developed the theory of the six necessary and sufficient conditions of the therapeutic relationship ( Rogers, 1957). Rogers gave the therapeutic relationship a wider definition where it also advocates providing a non-judgmental and non-directional therapeutic atmosphere where clients and therapists experience six therapeutic conditions, three of which are specific to the therapist: *empathic understanding, unconditional positive regard, and congruence*( Rogers, 1957) *.* Rogers’ therapeutic relationship conditions would be a complex of mutual, reciprocal, and dual interaction between mental health professionals and their clients ( Rogers, 1957; Barrett-Lennard, 2011; Murphy, Cramer, and Joseph, 2012). The six necessary and sufficient conditions are as follows ( Rogers, 1957, pp. 95): *1) Two persons are in psychological contact.2) The first, whom we shall term the client, is in a state of incongruence, being vulnerable or anxious. 3) The second person, whom we shall term the therapist, is congruent or integrated into the relationship. 4) The therapist experiences unconditional positive regard for the client. 5) The therapist experiences an empathic understanding of the client’s internal frame of reference and endeavours to communicate this experience to the client. 6) The communication to the client of the therapists’ empathic understanding and unconditional positive regard is to a minimal degree achieved.* By maintaining an experience of these conditions, a therapeutic relationship would benefit both clients and professional practitioners. The theory provides a useful framework for positioning the therapeutic relationship within psychiatry.

### Pedagogy in Psychiatry

The registered number of psychiatrists in South East Asia and Africa in 2014 has increased 25% since 2011, and the population of other mental health professionals, such as psychiatric nurses, has also grown by 37% ( World Health Organization, 2014, pp. 53). This increasing supply of mental health professionals reflects the growing prevalence of psychological distress in the general population. For example, in Taiwan in East Asia, it was reported that there were 1 in 4 people suffering from common mental health problems, such as depression and anxiety disorder (Fu *et al.,* 2013). The numbers of mental health service users are still multiplying rapidly ( World Health Organization, 2014). Therefore, providing effective training for mental health care becomes essential.

According to McCann *et al.* (2012), there are currently five approaches applied to develop a mental health professional covering pedagogical and quality aspects which including *professional development*, *topics and teaching methods*, *practice placements and supervised sessions,* and quality *assurance mechanisms.* Firstly, professional development providers students with current policy, services and practice development. Secondly, topics and teaching methods typically is a mixture of lecture format and other didactic methods, self-directed learning, and experiential learning. Thirdly, assessment of learning is used in assessing students on most courses. Fourthly, *practice placements and supervised sessions* require students to complete formal reports on placement supervision. Lastly, most courses use external examiners and formal feedback from students as the *quality assurance mechanisms* in the educational programmes ( McCann *et al.,* 2012). Hypothetically, mental health professional students acquire knowledge and skills to work in psychiatric healthcare and achieve the expectations of

*“working in partnership, respecting diversity, practicing ethically, challenging inequality, promoting recovery, identifying people’s need and strengths, user-centered care, making a difference, promoting safety and positive risk taking, and personal development and learning.”* ( McCann *et al.,* 2012, pp. 383).

The current medical education in the Mandarin-speaking world, and specifically Taiwan, has been influenced by Western culture due to the colonial and post-colonial history ( Cheng, 2011). For example, standard Taiwanese medical education is a 7-year Western education programme for doctors and a 4 or 5-year programme for other health professionals, such as nurses, dentists and psychotherapists. The curriculum of doctors’ education includes 2 years of pre-medical courses, 2.5-3 years of clinical course and 2.5-3 years of clerkship and internship. The programme of nurses and psychotherapists’ education comprises 1-1.5 years of integrated and basic clinical courses, and 2.5 years of practical placement in the medical settings ( Chou *et al.,* 2012). Before determining which subfield to serve in, medical students in Taiwan are required to follow the curriculum of medical education provided by the Ministry of Education ( Chou *et al.,* 2012). Following the principle of medical education given by the Accreditation Council for the Graduate Medical Education (ACGME) in the United States, the medical education in Taiwan has stated that the performance of health providers is determined by the framework of their competencies of medical knowledge, practice-based learning and improvement, professionalism, systems-based practice, patient care, and interpersonal and communication skills ( Rider and Nawotniak, 2010). Importantly, ACGME has emphasized the capabilities of taking care of patients and the abilities of interacting and communication should be taught to students within medical education ( Rider and Nawotniak, 2010). Acknowledging the professional practitioner-patient communication and the improvement of practitioners’ empathy skills are essential factors to influence patients’ prognosis and overcome the potential shortcomings associated with limited health-literacy capabilities ( Bendapudi *et al.,* 2006; Chu and Tseng, 2013). In this vein, the Ministry of Education in Taiwan has embraced the American and British standards of practitioner’s training to bring humanities into many areas of medical education, such as curriculum, licensing, student enrolment and the continuing education in health services ( Chiu and Tsai, 2009). There is not only the medical knowledge and practice to be gained through the 7-year programme, but also seminars about professional practitioner-patient relationship, such as empathic understanding of patients, communication skills and so on, which have to be learned during the pre-medical courses as part of the curriculum as required by the Ministry of Education in numerous of the medical schools ( Chou *et al.,* 2012; Chu and Tseng, 2013; Chiu and Tsai, 2009).

This challenges the current medical educators, clinical supervisors, preceptors and medical staffs in the psychiatric departments to consider revising current pedagogy and to design suitable educational programmes for mental health professional students. Although proactively applying a range of traditional and non-traditional learning methods to introduce health professional students to humanities courses, pre-medical and clinical courses ( Chou *et al.,* 2012; Chu and Tseng, 2013; Chiu and Tsai, 2009), the Ministry of Education Taiwan has requested more medical education concepts and models from other countries to be developed ( Chou *et al.,* 2012).

### Learning Interpersonal Skills in Psychiatry

Integrating the five pedagogical approaches of developing mental health professional students, using the *Helping Skills Model* becomes one of the common educational methods for mental health professional students ( Hill and Lent, 2006) and is considered an experiential learning method ( Slovák *et al.,* 2015). It integrates aspects from three traditional methods: *Human Relation Training* ( Carkhuff,1972), *Micro-Counselling* ( Ivey,1971), and *Interpersonal Process Recall* ( Kagan,1984). *Human Relation Training* (HRT) develops mental health professional students by rotating the roles of practitioner, client and observer during the interpersonal skills practice. *Micro-Counselling* (MC) focuses on developing specific interpersonal skills during role-play practice, with the tutor’s feedback and guidance *. Interpersonal Process Recall* (IPR) helps mental health professional students review their practice with their peers to reflect and deepen the understanding of what happened in the practice session ( Slovák *et al.,* 2015). The methods of the *Helping Skills Model* include *direct instruction* where the instructor gives information about target skills, *modelling* involves demonstration of specific skills and *feedback* from either the instructor or the other participants. These methods are often performed through the use of audio recording and video capture to facilitate practice ( Slovák *et al.,* 2015). Studies have found that video recording with peer feedback in real-time consultation is in favour of contemporary medical education with UK medical students (N=162) during training ( Eeckhout *et al.,* 2016), and also has indicated that self-reflecting on non-verbal behaviours, such as facial expression, un-purposive moment, body position, unnecessary giggling improved the communication skills of the medical students in the experimental group (N=134) with a significant change through using video training ( Park and Park, 2018).

mPath, a new technology software, advances the methods of the *Helping Skills Model*, as an innovative online software system developed specifically for mental health professional students. It provides the opportunity for a structured analysis of the mental health professional students’ practice sessions and aims to elicit and receive specific feedback from clients. The software has been produced by a cross-disciplinary team and offers the possibility for new technology to be added to existing methods of developing therapeutic relationship helping skills ( Slovák *et al.,* 2015; Murphy *et al.,* 2017; Murphy *et al.,* under review). It does this by deploying multiple tools to reflect on various aspects of peoples’ experiences. The entire process is all tightly linked with the video-recording of the session and designed to facilitate a time-efficient reflective feedback process.

To gain a deeper self-reflection on the session of mental health professional students’ education, mPath provides the opportunity of a structured reflection for students, allowing the opportunity to process a self-analysis whilst attaching specific thoughts or comments left as text annotations to the recorded video. mPathalso creates the space for interactions between the students as practitioners to collaborate with their clients. For example, the clients are able to share their thoughts, feelings and internal processes with the practitioners by giving feedback to them within the system. Furthermore, mPath offers the chance to enhance students’ therapeutic relationship competence by making annotation notes on the recorded practice videos, adapting to review different perspectives, requesting client’s feedback, deepening understanding of affect, and observing body movements ( Slovák *et al.,* 2015; Murphy *et al.,* 2017; Murphy *et al.,* under review).

## Methods

### Objective

The aim of the study was to test the effectiveness of a 3-day training on mental health professional students’ competence in the Rogerian therapeutic relationship skills. The objectives were to clarify the group’s and the individual’s baseline at the beginning of the study and evaluate the training effects at both group and individual level. To this end, the following research questions were posed.

• Is there any difference in baseline performance (pre-test, the first assessment) in terms of maturation by specialities education, such as nursing, occupational therapy, medicine, clinical psychology and others?
• If the participants obtained same baseline competence of Rogerian therapeutic relationship skills, do the control and experimental group show a significant difference in escalating behaviour before, at the end of and two weeks after the training intervention?
• If the participants obtained a difference in the baseline of Rogerian therapeutic relationship skills, does each group of each medical speciality show a significant difference in developing the competence before, at the end of and two weeks after the training intervention?
• Do the training results depend on the frequency of exposure in the experiential learning?

### Design

A pretest-posttest with follow-up within-and-between-group design was used. A cross-sectional analysis was performed to understand the participants’ competency in person-centred therapeutic relationship skills with and without the intervention to evaluate whether the therapeutic relationship skills could be learned in a short period of time and if the impact can be sustained at two weeks after the intervention ( Law, *et al.,* 2011; Barrett-Lennard, 2011; Barrett-Lennard 1962; Barrett-Lennard, 2018; Aukes et al., 2008; Nau, et al., 2010).

The Therapeutic Relationship Enabling Program (TREP) was designed as a process- and theory-oriented workshop where the participants were divided into two groups: the experimental group and control group ( Barrett-Lennard, 2011; Barrett-Lennard, 2018; Law, *et al.,* 2011). The control group did not receive the TREP intervention while the experimental group was exposed to the 3-day TREP workshop. Each group was presented with three assessments of the Mandarin-Chinese version of the Barrett-Lennard Relationship Inventory (B-L RI:MC) ( Liao, Murphy and Barrett-Lennard, 2018)used to rate their relationship competency, at the beginning, end of and two weeks after the intervention ( Barrett-Lennard, 1974/1975; Liao, Murphy and Barrett-Lennard, 2018). By comparing the multi-faceted outcomes of the within-and-between evaluations of the two groups, the effectiveness of the TREP would be considered.

### Sample and participants

Estimating the effective sample size for increasing the degree of confidence is preferred to recruit a statistical population on the basis of a relatively small amount of sample data ( Ellis, 2010; Kelley and Preacher, 2012). Cohen suggested that the effect sizes of small, medium and large are with a *d* of .2, .5, and .8 ( Hill and Lent, 2006; Cohen, 1988). According to the meta-analysis of the effectiveness of helping skills training surveyed by Hill and Lent, the aggregated effect size (d _+_) was .89 for conducting a training program ( Hill and Lent, 2006).

In 2016, there were 8,661 mental health professionals registered to work in psychiatric settings in Taiwan including hospitals, psychiatric clinics, day-care institutions and psychiatric nursing homes. There were 1,601 psychiatrists, 5,146 psychiatric nurses, 521 social workers, 685 clinical psychologists, 708 occupational therapists, 897 administers, 483 para-medical personnel, and 47 others worked in the licensed mental health professionals who have been working in the psychiatric settings and approximately 1,201 students studied in learning and practising psychiatric knowledge in medical schools (University Admissions Committee, 2014; Ministry of Health and Welfare, 2015). The target population is less than 2% of the entire population of Taiwan. Taiwanese mental health professional students are considered as a rare population. As the result of the estimation of an effective sample size, the minimum required sample size of the therapeutic relationship enabling program will approximately be 26 participants if the desired statistical power level is set as 0.8 and the probability level is 0.05 comparing to the whole population in Taiwan where there are 23,496,813 people. In this study, the aim was to have 3 workshops where 8 participants were in the experimental group in each workshop in order to reach 24 participants for the experimental group and 24 people for the control group sufficiently in this study. Therefore, it aimed to recruit more than 48 Taiwanese mental health professional students across two groups. The characteristics of the target group would be mental health professional students from medical-relevant professions in medical schools in Taiwan, such as psychiatrists, psychiatric nurses, clinical psychotherapists, occupational therapists, and the students practising/learning psychiatric knowledge in the psychiatric settings/schools and would be considered as a specific group providing psychiatric healthcare.

To identify prospective participants, snowball sampling was one of the suitable methods to access the target population ( Voicu and Babonea, 2011). The snowball sampling, also named referral sampling, is often used to target hidden populations, such as a particular group of people which is less than 2% of the entire population ( Voicu and Babonea, 2011). It allows researchers to use the participant’s social networks to refer to other prospective participants who could potentially contribute to the research ( Heckathorn,1997). However, this study aimed to recruit more than 24 Taiwanese mental health professional students for each experimental and control group to evaluate the change in the person-centred therapeutic relationship competence before, at the end and two weeks after the Therapeutic Relationship Enabling Programme.

Measuring Therapeutic Relationship in Psychiatry: Barrett-Lennard Relationship Inventory Mandarin-Chinese Version OS-40

The Barrett-Lennard Relationship Inventory (B-L RI) is a multiple-choice questionnaire, which is designed specifically for evaluating interpersonal relationships. It was developed by Barrett-Lennard when working with Carl Rogers in the University of Wisconsin where Rogers and his colleagues studied psychotherapy with people with a diagnosis of schizophrenia during the later 1950s and early 1960s( Thorne and Sanders, 2013, pp.112). Acknowledging the positive impact of Rogers’ theory, the B-L RI has been expanded and applied in evaluating different kinds of relationship. For example, therapist-client relationship, teacher-student relationship, family relationship, friendship, partnership, etc. B-L RI has been gradually adapted into different forms, such as 64-items and 40-items ( Liao, Murphy and Barrett-Lennard, 2018).

In this study, the Mandarin-Chinese version of Barrett-Lennard Relationship Inventory: Form Other Toward Self-40 (OS-40) will be used as the measure indicates promising reliability and construct validity in measuring therapeutic relationship conditions in Mandarin contexts ( Liao, Murphy and Barrett-Lennard, 2018). In Form OS-40, there are four dimensions in the Barrett-Lennard Relationship Inventory Mandarin-Chinese version, *level of regard, empathic understanding, unconditionality of regard* and *congruence*, and each dimension obtains 10 items (Barrett-Lennar, 2015; Liao, Murphy, & Barrett-Lennard, 2018). The *level of regard* refers to the affection of one person’s response to another, and it might embed positive or negative feelings ( Barrett-Lennard, 2015, pp. 11). The concept of *empathic understanding* is defined as one person is conscious of and aware of another ( Barrett-Lennard, 2015, pp.10). The definition of *unconditionality of regard* is given as the effective response and self-experiences of one person towards another ( Barrett-Lennard, 2015, pp. 11).

Finally, the concept of *congruence* is the consistency between the whole present experience and awareness. For example, a congruent person can be honest, sincere and direct to another without hesitation or feeling compelled during the communication ( Barrett-Lennard, 2015, pp. 11). Each item is rated on differing strengths of No or Yes in the range -3 to +3 ( Barrett-Lennard, 2015, pp. 26-34; 40-41). Each score of the scale would result in a possible range of -30 to +30. If avoiding negative values is necessary, it could add a constant of +30 to each obtained scale score to convert the scores with a possible range of 0 to 60 ( Barrett-Lennard, 2015, pp.122). Applying the standard scoring method of the 64-item scale in the 40-item scale, B-L RI:MC OS-40 would result in a score with a range of -30 (or -3 x10) to +30 (or +3 x 10) in each 10-item. A sub-score of 20 would indicate an average item score of 2 after converting the scores to the negatively worded items and represents an obvious affirmation of a person who experiences a positive therapeutic condition, like empathy, level of regard, etc. A sub-score of 15 would represent a less helpful relationship. Lastly, with a sub-score of 10, an assessment would discover a respondent experiencing a conceivably less than adequate level of therapeutic relationship ( Barrett-Lennard, 2015, pp. 39-42). However, the development and adaption of the Barrett-Lennard Relationship Inventory and its Mandarin-Chinese version were absolutely aligned with the core concept of person-centred therapeutic relationship ( Liao, Murphy and Barrett-Lennard, 2018).

Therefore, the Mandarin-Chinese version of the Barrett-Lennard Relationship Inventory (B-L RI:MC) was administered to the participants in both the control group and experimental group prior to the TREP as a pre-test evaluation. The post-test evaluation was conducted at the end of the TREP. The final assessment was performed 2 weeks after the TREP and before a qualitative interview took place ( Liao, Murphy and Barrett-Lennard, 2018; Messina, Sambin and Palmieri, 2013).

### Intervention: Therapeutic Relationship Enabling Programme

**Figure 1.**
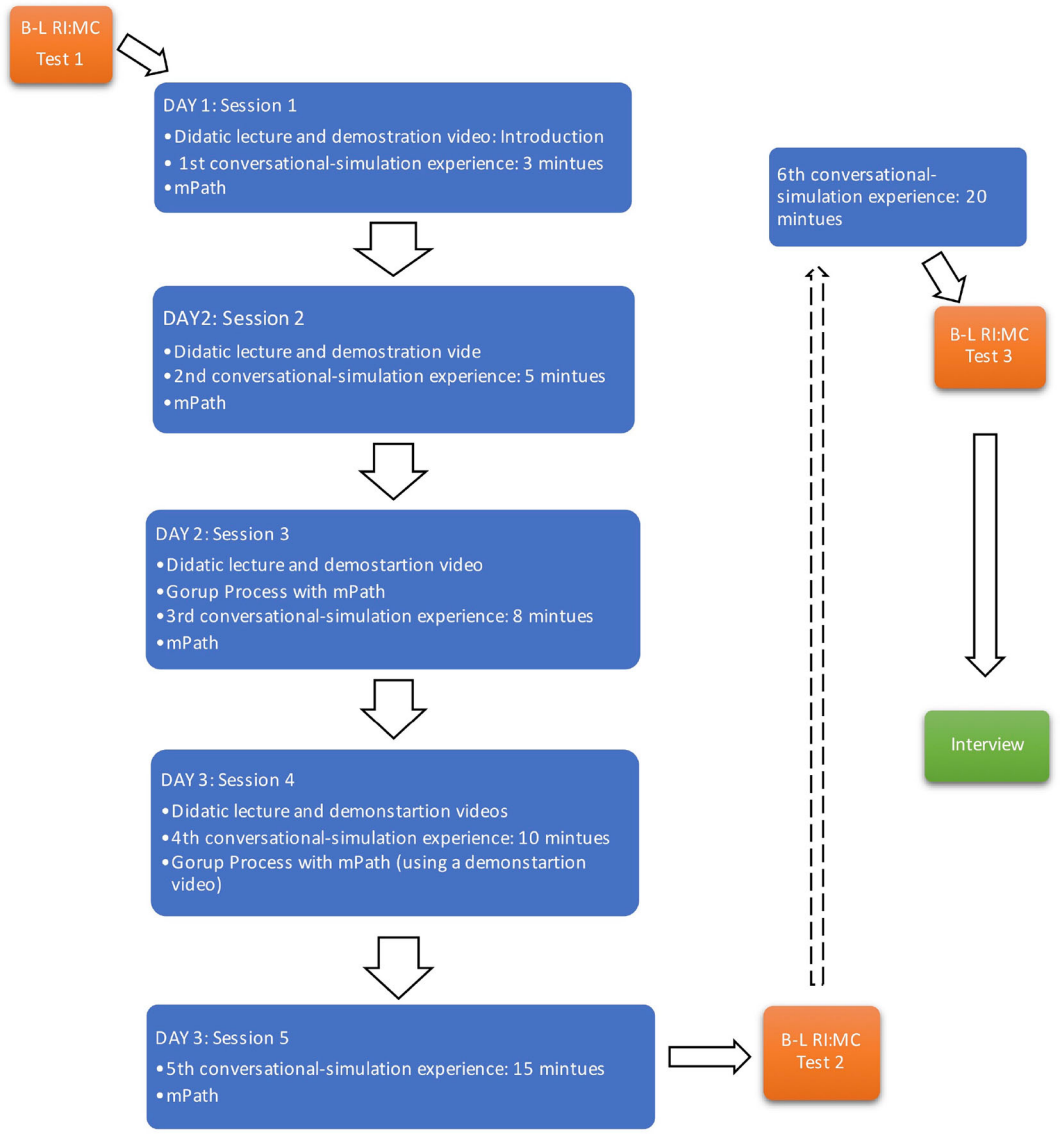
Procedure of Therapeutic Relationship Enabling Programme and the Quasi-Experiment

The TREP consisted of a 3-day workshop of 5 didactic lectures, 5 conversational-simulation experiential exercises, and 5 non-directive reflection and self-reflecting processes with technology support which was followed by one more conversational-simulation experience and a semi-structured interview after two weeks ( Figure 1) ( Aukes *et al.,* 2008; Bridges *et al.,* 2011; Cornelius-White *et al.,* 2018; Slovák *et al.,* 2015). There were 5 sessions designed in the intervention. Each session took 2 hours consisting of 1 didactic lecture, 1 conversational-simulation experience, 1 technological-based reflection with mPath and a break. The first and second assessments took place before and after the intervention to evaluate any change of participants’ performance and the effectiveness of the intervention. The final assessment was given after the 6 ^th^ conversational-simulation experience and before the interview to investigate the sustainability and development of the effect over time.

In the first session, an interactive activity was conducted to help learn about the different perspective of unconditional positive regard of each individual. The concepts of Rogers’ person-centredtherapy and therapeutic relationship were addressed by giving an introduction and a demonstration video. After giving a tutorial of mPath the participants, with their practice partner, were requested to videotape a 3-minute communicative practice and upload it to the software system, and then leave some annotations on mPath. Secondly, one of four dimensions of Rogerian therapeutic relationship was addressed in the course followed by another demonstration video. The participants then videotaped a 5-minute conversational-simulation experience with their peers, and then self-processed with the use of mPath. Thirdly, another dimension of Rogerian therapeutic relationship was given followed by a group process activity on mPathand an 8-minute conversational-simulation experience. The participants would self-analyse, share their thoughts and ask their partner for feedback in the system. Fourthly, the final two dimensions of Rogerian therapeutic relationship were introduced in mini-lecture format, and the participants had a 10-minute conversational-simulation experience. Finally, the participants had a 15-minute conversational-simulation experience with their partners, and then made annotation notes on the recorded practice videos, adapting to review different perspectives, requesting the partners’ feedback, deepening understanding of emotional affect, and observing their body movements.

The workshop was developed to fulfil three requirements. First of all, it aimed to ensure that Rogers’ person-centred theory was well-implemented in the workshop. For instance, the medical-educated trainer must specialize in the person-centred approach to counselling and psychotherapy in order to create a non-directive and non-judgmental atmosphere and adjust the training schedule flexible for the participants to experience the nature of person-centred approach and enhance their learning ( Rogers,1957; Aukes *et al.,* 2008; Barrett-Lennard, 2011; Barrett-Lennard, 2018). Secondly, it promoted the development of knowledge and competency of therapeutic relationship skills by giving the definitions of 4 conditions in Rogerian therapeutic relationship and then illustrating some of the examples of therapeutic competencies in the person-centred and experiential psychotherapy using video clips ( Rogers,1957; Freire, Elliott and Westwell, 2014; Barrett-Lennard, 2011; Barrett-Lennard, 2018). Lastly, it delivered the theoretical, conception and empirical evidence for each element in the TREP. For example, the research ethics and rationale of the training workshop, the results of the validation of Barrett-Lennard Relationship Inventory Mandarin-Chinese version, and the publications of the development and application of the helping learning technology mPath ( Slovák *et al.,* 2015; Barrett-Lennard, 1962; Barrett-Lennard, 2018; Liao, Murphy and Barrett-Lennard, 2018).

## Results/Analysis

### Demography of Participants

59 mental health professional students, with a mean of age 24 in Taiwan were recruited to this study, including 26 people in the experimental group and 33 people in the control group. 50 of 59 participants were female, and 9 of them were male ( Table 1). 30 were nursing students, 6 were studying in occupational therapy, 10 were medical students, 10 were in clinical psychology, and 3 were students in other mental-health-relevant professions. All participants had completed psychiatric-related lectures in medical schools, such as neurology, psychology, psychiatric nursing, psychotherapy, psychological occupational therapy, however, they reported little or no prior exposure to psychiatric settings.

In the experimental group, the participants were requested to participate in the TREP in pairs to evaluate the change of the therapeutic relationship competence of their partners. There were 24 females and 2 males with a mean of age 22 years. 15 of them were trained in nursing, 5 in occupational therapy, 4 in medicine, 2 in clinical psychology, and none in other mental-health-relevant professionals. 18 of 26 participants in the experimental group were rating a peer-relationship with 3 to 5 years. 8 of them were rating relationships with either 1 to 3 years or more than 5 years.

On the other hand, the controls were recruited through snowball sampling. They were required to complete 3 evaluations of the therapeutic relationship competence of a mental health professional student who did not experience the Therapeutic Relationship Enabling Program. With a mean of age 24 years, the majority of the controls were female and nursing students. 26 females and 7 males. There were 15 controls studying in nursing, 1 in occupational therapy, 6 in medicine, 8 in clinical psychology and 3 in other mental-health-relevant specifies. In terms of years of the evaluated peer-relationship, the relationship with 3 to 5 years was mostly rated, and then the one with 1 to 3 years went second, and finally one with more than 5 years was the last. None of the evaluated relationships were less than 12 months.

The result of the Mandarin-Chinese version of Barrett-Lennard Relationship Inventory: Form OS-40 (B-L RI:MC OS-40), 59 participants rated a total mean score of 43.20 and standard deviation of 23.10 in the scale where the sub-scales of *level of regard*(M= 17.49, SD= 6.64), *empathic understanding*(M= 9.92, SD= 8.73), *unconditionality of regard*(M= 2.14, SD= 6.20) and *congruence*(M= 13.66, SD= 8.74) were embedded ( Table 4). The result above was aligned with other samples where the average score of *level of regard* tended to be higher than other sub-scales and the scores of *unconditionality of regard* were the lowest one in the scale ( Barrett-Lennard, 2015, pp. 41).

**Table 1.** Characteristic of the Sample of Mental Health Professional Students in Taiwan

Characteristic	Experimental Group (N=26)		Control Group (N=33)		Total (N=59)	
	N	%	N	%	N	%
Age (years)						
*20-29*	24	92.30	27	81.82	51	86.44
*30-39*	1	3.85	3	9.09	4	6.78
*40-49*	1	3.85	1	3.03	2	3.39
*>50*	0	0.00	2	6.06	2	3.39
*Means (M)*	22.96		25.21		24.22	
*Standard Deviations (SD)*	5.64		8.02		7.10	
Gender						
*Male*	2	7.70	7	21.20	9	15.30
*Female*	24	92.30	26	78.80	50	84.70
*Other*	0	0.00	0	0.00	0	0.00
Specialties						
*Nursing*	15	57.70	15	45.50	30	50.80
*Occupational Therapy*	5	19.20	1	3.00	6	10.20
*Medicine*	4	15.40	6	18.20	10	16.90
*Clinical Psychology*	2	7.70	8	24.20	10	16.90
*Others*	0	0.00	3	9.10	3	5.10
Year of Evaluated Peer-relationship						
*Less than 6 months*	0	0.00	0	0.00	0	0.00
*6-12 months*	0	0.00	0	0.00	0	0.00
*1-3 years*	4	15.38	12	36.36	16	27.10
*3-5 years*	18	69.23	15	45.45	33	55.90
*More than 5 years*	4	15.38	6	18.18	10	16.90

### Examining Participants’ Baseline Competence of Rogerian Therapeutic Relationship: Between Specialties

A one-way between subject ANOVA was conducted to compare the baseline competence rating of therapeutic relationship skills of the mental health professional students across specialities: nursing, occupational therapy, medicine, clinical psychology and others ( Table 2; Table 3). There was no significant difference in amount of baseline competence on participants’ specialties at the p> .05 level for the five specialties [F (4, 54) = .28, *p=* .887]. This tests the null hypothesis that the error variance of the dependent variable is equal across groups. Furthermore, the baseline competence of therapeutic relationship of participants who specialize in nursing (N=30, M= 46.43, SD= 21.55) did not significantly differ from the participants who were studying in occupational therapy (N=6, M= 48.17, SD= 23.77), medicine (N=10, M= 43.30, SD= 25.54), clinical psychology (N=10, M= 32.30, SD= 25.56) and others (N=3, M= 37.00, SD= 23.90) ( Table 2; Table 3). In the table of the pairwise comparisons between specialities, with p > .05, it shows that there was no significant difference of the therapeutic relationship competence between each speciality of the mental health professional students. For example, the *p* values resulted in .868 with the occupational therapy students, .713 with the medical students, .102 with the clinical psychology students, and .506 with the students in other subjects if pair-comparing with the nursing students. With the occupational therapy students, there was no significant difference between the students in medicine with a *p* value of .687, clinical psychology with a *p value* of .192 and other subjects with a *p* value of .500. The medicine and clinical psychology students also showed no statistically significant difference from each other with a *p* value of .295 ( Table 3).

**Table 2.** Descriptive Statistics Between Specialties of Participants Dependent Variable

Specialities	Mean	Std. Deviation	N
Nursing	46.43	21.548	30
Occupational Therapy	48.17	23.769	6
Medicine	43.30	25.539	10
Clinic Psychology	32.30	25.561	10
Others	37.00	23.896	3
Total	43.20	23.099	59

**Table 3.** Pairwise Comparisons Between Specialties Dependent Variable

(I) Specialities		Mean Difference (I-J)	Std. Error	Sig. a	95% Confidence Interval for Difference a	
					Lower Bound	Upper Bound
Nursing	Occupational Therapy	-1.733	10.396	.868	-22.577	19.110
	Medicine	3.133	8.489	.713	-13.885	20.152
	Clinical Psychology	14.133	8.489	.102	-2.885	31.152
	Others	9.433	14.077	.506	-18.789	37.655
Occupational Therapy	Nursing	1.733	10.396	.868	-19.110	22.577
	Medicine	4.867	12.005	.687	-19.201	28.934
	Clinical Psychology	15.867	12.005	.192	-8.201	39.934
	Others	11.167	16.438	.500	-21.790	44.123
Medicine	Nursing	-3.133	8.489	.713	-20.152	13.885
	Occupational Therapy	-4.867	12.005	.687	-28.934	19.201
	Clinical Psychology	11.000	10.396	.295	-9.843	31.843
	Others	6.300	15.303	.682	-24.381	36.981
Clinical Psychology	Nursing	-14.133	8.489	.102	-31.152	2.885
	Occupational Therapy	-15.867	12.005	.192	-39.934	8.201
	Medicine	-11.000	10.396	.295	-31.843	9.843
	Others	-4.700	15.303	.760	-35.381	25.981
Others	Nursing	-9.433	14.077	.506	-37.655	18.789
	Occupational Therapy	-11.167	16.438	.500	-44.123	21.790
	Medicine	-6.300	15.303	.682	-36.981	24.381
	Clinical Psychology	4.700	15.303	.760	-25.981	35.381

*Based on estimated marginal means*

a. *Adjustment for multiple comparisons: Least Significant Difference (equivalent to no adjustments).*

### Examining Participants’ Baseline Competence of Rogerian Therapeutic Relationship: Between Conditions

An independent-sample t-test was conducted to compare participants’ competence of therapeutic relationship in the intervention and non-intervention conditions. There was not a significant difference in the total score of Barrett-Lennard Relationship Inventory Mandarin-Chinese version for the experimental group (M= 44.50, SD= 18.99) and the control group (M= 42.18, SD= 26.14); *t*(57) = .38, *p=* .71. These results suggest that the participants in both experimental and control group obtained similar baseline competence of Rogerian therapeutic relationship skills at recruitment ( Table 4; Table 5). A report of Box’s test of Equality of Covariance Matrices also indicated the null hypothesis that the observed covariance matrices of the dependent variables ( *p=* .024) are equal across groups. Therefore, it can be concluded that there was no statistically significant difference in Rogerian therapeutic relationship competence between participants and the two groups.

**Table 4.** Groups Statistics BEFORE the Intervention

Measure	Group	N	Mean	Std. Deviation	Std. Error Mean
Total Score of B-L RI:MC	Experimental Group	26	44.50	18.98	3.72
	Control Group	33	42.18	26.14	4.55
	Experimental + Control Group	59	43.20	23.10	3.01
*Level of Regard*	Experimental Group	26	17.92	5.97	1.17
	Control Group	33	17.15	7.19	1.25
	Experimental + Control Group	59	17.49	6.64	0.86
*Empathic Understanding*	Experimental Group	26	10.38	6.93	1.36
	Control Group	33	9.55	10.01	1.74
	Experimental + Control Group	59	9.92	8.73	1.14
*Unconditionality of Regard*	Experimental Group	26	2.35	6.21	1.22
	Control Group	33	1.97	6.29	1.09
	Experimental + Control Group	59	2.14	6.20	0.81
*Congruence*	Experimental Group	26	13.85	8.55	1.68
	Control Group	33	13.52	9.01	1.57
	Experimental + Control Group	59	13.66	8.74	1.14

**Table 5.** Independent Samples Test of Experimental and Control Group BEFORE the Intervention

Measure		Levene’s Test for Equality of Variances		*t*	*df*	Sig. (2-tailed)	*t*-Test for Equality of Means		95% Confidence Interval of the Difference	
		*F*	Sig.				Mean Difference	Std. Error Difference	Lower	Upper
Total Score of B-L RI:MC	Equal variances assumed	3.07	0.09	0.38	57.00	0.71	2.32	6.10	-9.90	14.54
	Equal variances not assumed			0.39	56.67	0.70	2.32	5.88	-9.46	14.09
*Level of Regard*	Equal variances assumed	1.48	0.23	0.44	57.00	0.66	0.77	1.75	-2.74	4.28
	Equal variances not assumed			0.45	56.83	0.65	0.77	1.71	-2.66	4.20
*Empathic Understanding*	Equal variances assumed	3.58	0.06	0.36	57.00	0.72	0.84	2.31	-3.78	5.46
	Equal variances not assumed			0.38	56.17	0.71	0.84	2.21	-3.59	5.27
*Unconditionality of Regard*	Equal variances assumed	0.26	0.61	0.23	57.00	0.82	0.38	1.64	-2.91	3.66
	Equal variances not assumed			0.23	54.12	0.82	0.38	1.64	-2.91	3.66
*Congruence*	Equal variances assumed	0.00	0.97	0.14	57.00	0.89	0.33	2.31	-4.30	4.96
	Equal variances not assumed			0.14	55.01	0.89	0.33	2.30	-4.27	4.93

### Changes and Influences on Learning Progress

A one-way repeated measures ANOVA was conducted to compare the change of the competence of Rogerian therapeutic relationship on mental health professional students, before, at the end and 2 weeks after the Therapeutic Relationship Enabling program. There was a significant change of the competence, Wilk’s Lambda= .731, *F*(2, 56) = 10, *p=* .000 ( Table 6). It reported that the participants’ competence of Rogerian therapeutic relationship had a significant difference overall.

**Table 6.** Multivariate Tests of Repeated Measure One-Way ANOVA

Effect		Value	*F*	Hypothesis *df*	Error *df*	Sig.	Partial Eta Squared
Before_At theEnd_Two Weeks After	Pillai’s Trace	.269	10.286 ^b^	2.000	56.000	.000	.269
	Wilks’ Lambda	.731	10.286 ^b^	2.000	56.000	.000	.269
	Hotelling’s Trace	.367	10.286 ^b^	2.000	56.000	.000	.269
	Roy’s Largest Root	.367	10.286 ^b^	2.000	56.000	.000	.269
Before_At theEnd_Two Weeks After * Condition	Pillai’s Trace	.289	11.396 ^b^	2.000	56.000	.000	.289
	Wilks’ Lambda	.711	11.396 ^b^	2.000	56.000	.000	.289
	Hotelling’s Trace	.407	11.396 ^b^	2.000	56.000	.000	.289
	Roy’s Largest Root	.407	11.396 ^b^	2.000	56.000	.000	.289

a *Design: Intercept + ConditionWithin Subjects Design: Before_At theEnd_Two Weeks After*

b *Exact statistic*

To measure the within-subjects effects in the test ( Table 7), the result of Greenhouse-Geisser correction reported that when using an ANOVA with repeated measures with a Greenhouse-Geisser correction, the mean scores for the evaluations were statistically significantly different ( *F*(1.709, 97.386) = 14.721, *p=* .000) between time points. It also indicated a significant difference between the experimental and control group with the mean scores F(1.709, 97.386) = 16.38, *p=* .000. Thus, it can say that those attending the TREP showed a statistically significant change in the level of competence in developing the therapeutic relationship with others over time.

**Table 7.** Tests of Within-Subjects Effects

Sources		Type III Sum of Squares	*df*	Mean Square	*F*	Sig.	Partial Eta Squared
Before_At theEnd_Two Weeks After	Sphericity Assumed	2600.023	2	1300.011	14.721	.000	.205
	Greenhouse-Geisser	2600.023	1.709	1521.787	14.721	.000	.205
	Huynh-Feldt	2600.023	1.787	1455.001	14.721	.000	.205
	Lower-bound	2600.023	1.000	2600.023	14.721	.000	.205
Before_At theEnd_Two Weeks After * Condition	Sphericity Assumed	2894.644	2	1447.322	16.389	.000	.223
	Greenhouse-Geisser	2894.644	1.709	1694.228	16.389	.000	.223
	Huynh-Feldt	2894.644	1.787	1619.874	16.389	.000	.223
	Lower-bound	2894.644	1.000	2894.644	16.389	.000	.223
Error (Before_At theEnd_Two Weeks After)	Sphericity Assumed	10067.605	114	88.312			
	Greenhouse-Geisser	10067.605	97.386	103.378			
	Huynh-Feldt	10067.605	101.857	98.841			
	Lower-bound	10067.605	57.000	176.625			

**Figure 2.**
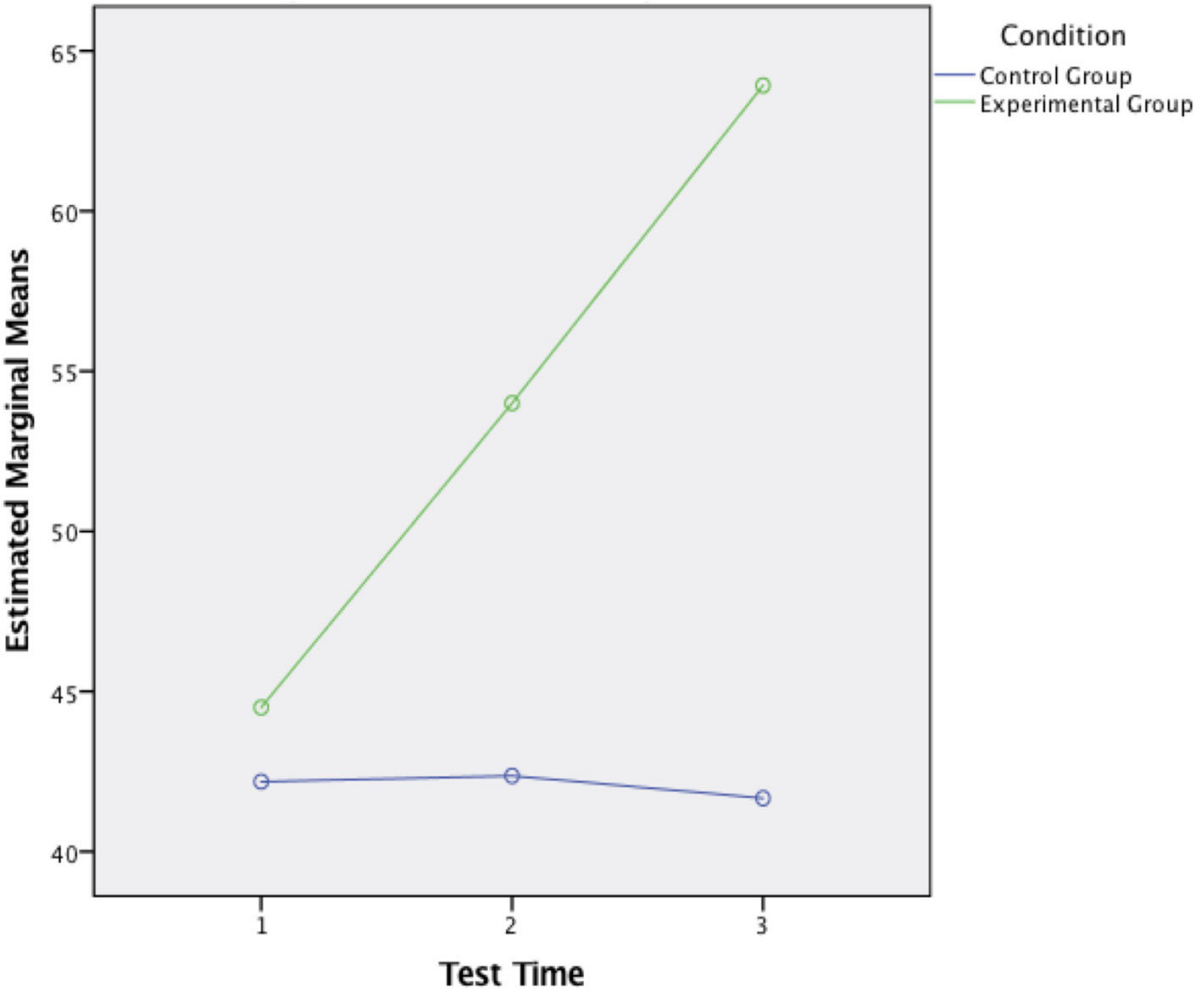
Estimated Marginal Means of Score

Three paired samples t-tests were used to make post hoc comparisons between each evaluation ( Figure 2). In the experimental group, a first paired sample t-test indicated that there was a significant difference between the mental health professional students’ competence of Rogerian therapeutic relationship before (M= 44.50, SD= 18.98) and at the end (M= 54, SD= 19.05) of the intervention; *t* (25) = -3.51, *p=* .002. A second paired samples t-test indicated that there was a significant difference between the mental health professional students’ competence of Rogerian therapeutic relationship at the end of (M= 54, SD= 19.05) and two weeks after (M= 63.92, SD= 17.83) the intervention; *t*(25) = -.348, *p=* .002. A third paired samples t-test indicated that there was a significant difference between the mental health professional students’ competence of Rogerian therapeutic relationship before (M= 44.50, SD= 18.98) and two weeks after (M= 63.92, SD= 17.83) the intervention; *t*(25) = -5.12, *p=* .000 ( Table 8). Hence, it can be concluded that Therapeutic Relationship Enabling Program initiates the participants’ therapeutic relationship competence in a short-term period, furthermore, the competence remains and increases while the participants were no longer exposed to the training environment.

**Table 8.** Descriptive Statistics and Paired Samples T-Test of Experimental Group and Control Group

Groups	Descriptive Statistics			Paired Differences									
	Test Time	Mean	SD			Mean	SD	Std. Error Mean	95% Confidence Interval of the Difference		*t*	*df*	Sig. (2-tailed)
									Lower	Upper			
**Experi-mental Group (N=26)**	*Before*	44.50	18.98	**Pair 1**	*Before - At the End*	-9.500	13.785	2.703	-15.068	-3.932	-3.514	25	.002
	*At the End*	54.00	19.05	**Pair 2**	*At the End - Two Weeks After*	-9.923	14.555	2.854	-15.802	-4.044	-3.476	25	.002
	*Two Weeks After*	63.92	17.83	**Pair 3**	*Before - Two Weeks After*	-19.423	19.346	3.794	-27.237	-11.609	-5.119	25	.000
**Control Group (N=33)**	*Before*	42.18	26.14	**Pair 1**	*Before - At the End*	-.182	9.551	1.663	-3.568	3.205	-.109	32	.914
	*At the End*	42.36	27.51	**Pair 2**	*At the End - Two Weeks After*	.697	9.729	1.694	-2.753	4.147	.412	32	.683
	*Two Weeks After*	41.67	24.29	**Pair 3**	*Before - Two Weeks After*	.515	12.314	2.144	-3.851	4.881	.240	32	.812

In contrast, the report of three paired samples t-test in the control group showed no significant difference between each evaluation. A first paired sample t-test indicated that there was no significant difference between the mental health professional students’ competence of Rogerian therapeutic relationship before (M= 42.18, SD= 26.14) and at the end (M= 42.36, SD= 27.51) of the intervention; *t*(33) = -.109, *p=* .914. A second paired samples t-test indicated that there was no significant difference between the mental health professional students’ competence of Rogerian therapeutic relationship at the end of (M= 42.36, SD= 27.51) and two weeks after (M= 41.67, SD= 24.49) the intervention; *t*(32) = .412, *p=* .683. A third paired samples t-test indicated that there was no significant difference between the mental health professional students’ competence of Rogerian therapeutic relationship before (M= 42.18, SD= 26.14) and two weeks after (M= 41.67, SD= 24.49) the intervention; *t*(32) = .240, *p=* .812 ( Table 8). Thus, it can conclude that the level of therapeutic relationship competence of the participants who were not exposed to the Therapeutic Relationship Enabling Program showed no significant difference before and at the end of the intervention. Nevertheless, instead of remaining at the level of competence, there was a decrease in the participants’ therapeutic relationship competence appeared over time.

## Discussion

The goal of this study was to examine the expectation that a theoretically informed, experientially-based training workshop with the use of a theory-designed technology would enhance mental health professional students’ learning on therapeutic relationship skills. The workshop, Therapeutic Relationship Enabling Programme (TREP), has given the participants a person-centred-theory-based definition of therapeutic relationships in mental healthcare and provided an interactive method for fostering self-reflection in mental healthcare training. These findings provide some evidence that a short-period training is suitable to enhance Taiwanese mental health professional students’ competence in therapeutic relationships, and after attending a person-centred learning workshop, students’ competence remained and further increased.

### Participants’ Baseline Competence of Therapeutic Relationship

The study showed the participants obtained an identical baseline of therapeutic relationship competence regardless of which group they were in the Therapeutic Relationship Enabling Programme and their specialities in medical schools. As the result in this study, the statistical report with p&gt; .05 indicated that the students who are specializing in nursing, occupational therapy, medicine, clinical psychology and other subjects had no difference significantly to each other before they participated in the Therapeutic Relationship Enabling Programme ( Table 3). It showed that the current medical education in Taiwan had provided mental health professional students with a similar insight of therapeutic relationship with a mean score 43.20 and standard deviation 23.10 regardless various pedagogical approaches in the educational programs ( Table 2). Therefore, it could be concluded that although the participants were voluntarily recruited and snowball-sampled from 7 medical schools in Taiwan, they nevertheless shared a similar level of understanding the therapeutic relationship.

The findings also showed although the speciality of participants, including the experimental and control group, were very, there was no significant difference of therapeutic relationship competence between the nursing (N=30), medicine (N=10), clinical psychology (N=10), occupational therapy (N=6) and others (N=3) students before the intervention. In Table 3, the *p* values of each comparison between the groups of nursing, occupational therapy, medicine, clinical psychology and other students all resulted in more than .05 which accepted the null hypothesis of no difference of the baseline therapeutic relationship competence between each speciality. It also evidenced that the overall mental health professional students in Taiwan would demonstrate a similar level of therapeutic relationship competence regardless of their specialities, educational pedagogies, and learning environments. Taiwanese medical education is considered as a cluster of a medical knowledge framework, problem-based learning and improvement and professionalism, systems-based practice, patient care, and interpersonal communication skills (Albanese, 2000; Cheng, 2001; Chiu and Tsai, 2009; Chou *et al.,* 2012). It would advantage the health professional students achieve an adequate level of therapeutic relationship competence and skills in mental healthcare effectively. However, its disadvantage might result in a consequence for the future mental health professionals responding to their clients/patients non-organically and inflexibly while having ineffective learning in an ossified educational environment.

Taiwanese mental health professional students&rsquo; level of experiencing the 4 domains of the therapeutic relationship in practice varied. First of all, with the mean score of 17.49 (above a score of 15) in the *level of regard* sub-scale before the intervention, the score suggests that participants perceived a minimal degree of *level of regard* with their practice peers before the workshop intervention. From this, it is possible to consider that mental health professional students might deliver a lower but not least satisfying level of regard towards prospective clients/patients in a therapeutic relationship. Thus, it could be suggested to further investigate how the existing communication skills training supports delivering a level of positive regard towards patients Secondly, the mean score of *empathic understanding* 9.92 was slightly less than a standard score of 10 which indicated that the participants experienced on average a minimally lower level of being empathically understood. Theoretically, mental health professional would be expected to empathize with their clients/patients at an adequate level in a therapeutic relationship ( Hung, Huang and Lin, 2009; Ng and Chan, 2000; Parker and Leggett, 2012; Rogers *et al.,* 1967; Goering and Stylianos,1988; Goering and Stylianos,1988). The Ministry of Education Taiwan has applied the American and British standards of practitioners training in the pedagogy of medical education for many years, for example, introducing humanistic lectures and seminars alongside with the clinical and non-clinical courses ( Chou *et al.,* 2012). However, the result of this study has discovered that there is still a way to go for Taiwan mental health professional students to develop and deliver a satisfactory level of empathic understanding towards their clients/patients. It has raised the question as to whether the current pedagogy favourably accommodated the demand in psychiatry?

Besides the traditional learning methods, could technology facilitate mental health professional students to gain more insight into of therapeutic relationship? Hence, re-examining and evaluating the advantages and disadvantages of the current courses content and context on communication skills in medical education would be one of the suggestions for mental health professional students acquiring communication competency. Furthermore, the mean score of the sub-scale *unconditionality of regard* 2.14 endorsed the results of other studies where the score of *unconditionality of regard* usually is rated lowest across the Barrett-Lennard Relationship Inventory ( Barrett-Lennard, 2015, pp. 41). It would be worth exploring possible causes of the low rating for Taiwanese mental health professional students. For example, from educational programs, cultural perspective, social context, etc. Lastly, the mean score of the *congruence*s sub-scale 13.66 was lower than a mean score of 15 indicated the participants experiencing a minor level of fruitful helping relationship with their peers. This could raise the assumption that clients/patients might not perceive consistently-positive attention and attitude from Taiwanese mental health professional students in a therapeutic relationship. On the other hand, Taiwanese mental health professional students might not show expectedly sincere and direct feelings when communication takes place so that their clients/patients might be feeling hesitation or obliged to act a certain way in a therapeutic relationship.

### Changes in Competence of Therapeutic Relationship on Learning Progress

The statistics reported in Table 6, 7 and 8 indicated a positive outcome of the intervention where the participants who attended the program have gained competence in the therapeutic relationship, and the ones who did not participate in the workshop remained at the same level of competence. In contrast, members in the control group encountered a decrease two weeks after the intervention while the experimental group participants&rsquo; competence increased continuously and organically ( Figure 2).

For the experimental group, the result showed a significant change in the participants&rsquo; competence of Rogerian therapeutic relationship at the end of the intervention with a *p* value of .00 which rejected the null hypothesis that there was no significant difference between each measurement. Pairing the changes between the time point *before* (M= 44.50, SD= 18.98) and *at the end of the intervention* (M= 54.00, SD= 19.05), the mean scores statistically increased with a *p* value of .002 which represents that the participants in the experimental group had gained a statistically significant increase in level of competence of therapeutic relationship. It also evidenced that the effectiveness of a short theory-based training workshop with the use of a theory-designed technology is significant and sufficient for mental health professional students in Taiwan to develop their clinical communication skills in practice. The TREP can be considered an innovative 3-day workshop that facilitates learners enhancing the understanding and competence of Rogerian therapeutic relationship experientially with the usage of an interactive interface mPath, in a person-centred design curriculum. The program allows learners to gain ownership over their learning experience and modify their learning approaches ( Rousmaniere, 2014; Forbes *et al.,* 2016; Kolb, 1984). For example, the learners inspiringly inquired to have a group analysis on a demonstration clip on mPath. Everyone was encouraged to create tracks of annotation, such as body languages and affection of emotion, together as a group. Through the group dynamics, the discussions and debates between each individual imprinted and clarified the notion of the conditions of a Rogerian therapeutic relationship in practice, meanwhile, the individual voice about the understanding of Rogerian therapeutic relationship was heard and their doubts about their own confidence in developing a therapeutic relationship with clients/patients was reduced ( Barrett-Lennard, 2018; Murphy *et al.,*under review). It might be worth to further investigate the participants&rsquo; experiences of learning the Rogerian therapeutic relationship and any highlights in the workshop. These further research studies might shed light on to further develop therapeutic relationship training in psychiatry in medical schools.

Surprisingly, the therapeutic relationship competence of the experimental group seems not only to have been sustained but also to have increased over the following two weeks after the short-term intervention. In Table 8, the data discovered that there was a continual growth of therapeutic relationship competence with a *p* value of .002 in the paired sample t-test between the assessments of *at the end* and *two-week-after.* In Figure 2, it also displayed a climbing growth process with mean scores of 44.50, 54.00 and 63.92 in three test times. On this evidence, it could be said that the short-term theory-based Therapeutic Relationship Enabling Programme with new technology involved has brought substantial concordance and appreciable changes for the experimental group. In contrast, the development of therapeutic relationship competence of the control group members showed a reverse and decreasing mean scores of 42.18 ( *p=* .914), 42.36 ( *p=* .683) and 41.67 ( *p=* .812) in three evaluations where they did not attend the workshop ( Figure 2; Table 8).

Having established the effects of the TREP workshop, further research is now required to investigate in more detail the individual components of the learning process. For example, an exploration investigates which learning experience made the change in competence during and after the workshop: didactic lectures, demonstration videos, conversational-simulation experience, mPath or other? Of which aspects that the participants experienced enabled them to increase the therapeutic relationship competence in a continuous manner? Was there any effect or impact that occurred to them in terms of experienced relational and personal change during or after the workshop? If there was a significant change in attitudes and perspectives as a mental health professional, why and how did it happen? The results of this study have clearly created more research questions to investigate. Doing so will carry our understanding forward about the suitability and possibility of modifying current teaching and learning approaches in medical education for psychiatry and the applicability of introducing innovative pedagogies for mental health professional students.

## Conclusion

The study supports the assumption that the definition of Carl Rogers’ person-centred therapeutic relationship could be accepted and applied in psychiatric contexts in medical education. The statistical data reports that the expectation that a theory-based training workshop with the use of a theory-designed technology enhanced mental health professional students’ learning on the therapeutic relationship was satisfied. A significant growth curve in therapeutic relationship competence of the experimental group was found in the follow-up assessment meaning that the competence has not only sustained but also grown organically after a short exposure of person-centred experientially learning.

A further investigation on how Taiwanese mental health professional students learnt Rogerian therapeutic relationship in the intervention, in which sessions that they came to a realization of the nature of Rogerian therapeutic relationship personally and what enhanced the learning experience the most might be recommended. Although there are various courses on humanity have been given in medical education, for example, seminars, group discussions and problem-based learning, Taiwanese mental health professional students’ baseline competence of therapeutic relationship still reached a degree of less satisfaction. However, the current medical pedagogy has been delivered in medical schools for years and undeniably benefited public health, for instance, epidemic prediction and prevention, cure rate improvement, birth control and so on. Regarding the increasing population of psychological distress and the demand of mental health care, looking into the impact on learning therapeutic relationship that the Therapeutic Relationship Enabling Programme (TREP) has brought the participants, and evaluate what works for mental health professional students in future practice might be beneficial.

The advantage of this study was the variety of mental health professional students’ speciality from seven medical schools in Taiwan, for example, student nursing, occupational therapy, clinical psychology and medical students, and it could reflect the current mental health education in Taiwan. However, the unequal distribution of the characteristics of target population could still be argued as a possible week point in this study even though the number of mental health professional students in the control group is more than the number in the experimental group.

Regarding the other limitations of this study, firstly, the study findings could have been strengthened with the introduction of a follow-up evaluation after 3 months to evidence a sustained efficacy and change in participants’ behaviour and/or outcomes. Future studies ought to factor this element into their design. Additionally, qualitative research conducted in which the participants were interviewed to investigate into the depth of learning would also help medical educators grasp how the therapeutic relationship skills are acquired in such a short period as shown in this study. Secondly, there were less than 10 male mental health professional students recruited in the experimental and control group where 50 female students participated in the study. A compared analysis on therapeutic relationship could be conducted to discover the initial understanding and competence of therapeutic relationship between genders, and furthermore, to look into if there is any growth or decrease of the competence at the end and two weeks after the intervention. However, the difference of numbers of genders did not influence the overall results of studying into the baseline competence and increase/decrease in a therapeutic relationship.

Thirdly, the number of mental health professional students’ speciality could be recruited equally. In this study, there were 15 nursing students participated in both the experimental and control group, whereas 5 occupational therapy students exposed in the intervention and 1 in the control group. Unquestionably, the empirical findings discovered there was no specific correlation between the baseline competence of the therapeutic relationship and the participants’ specialities. However, if there were an equal number of students in each speciality individually which would possibly enable to explore a further study on the growth/decrease of therapeutic relationship competence in each speciality for medical education. Lastly, a follow-up study could be conducted systemically at a later time point, such as six months and one year in medical education, or even one year after exposing in psychiatric settings as staffs.

The results of this study have shown the participants in the experimental group a growth of therapeutic relationship competence two weeks after the intervention, nevertheless, it might be worth to study on to what extent the effect of the intervention continues, whether it increase/ decrease at a later time, and what the factors are if the level of therapeutic relationship does increase/decrease.

## Take Home Messages

• Therapeutic Relationship Enabling Programme (TREP) has brought the participants impact on the acquisition of the therapeutic relationship.
• The study supports the assumption that the definition of Carl Rogers’ person-centred therapeutic relationship could be accepted and applied in medical education, especially the psychiatric contexts.
• The statistical data reports that the expectation that a theory-based training workshop with the use of a theory-designed technology enhanced the mental health professional students’ learning on the person-centred therapeutic relationship was satisfied.
• A significant growth curve in the therapeutic relationship competence of the experimental group was found in the follow-up assessment which indicated the competence has not only sustained but also grown organically after a short exposure of person-centred experientially learning.
• Interviews have been further conducted to explore the participants’ learning experiences before, during and after the TREP and investigate in the organic growth of the learner’s therapeutic relationship competence.

## Notes On Contributors

*Faith Liao* (ORCID https://orcid.org/0000-0002-8118-2998), PhD Candidate in the cross-disciplinary studies of Medical Education, Person-Centred Experimental Approach and technology-enhanced human relations at the University of Nottingham, United Kingdom. Faith holds BSc in Nursing and MA in Philosophy of Mind and Cognition, and Faith is also Registered Nurse in Taiwan.

*David Murphy* (ORCID https://orcid.org/0000-0003-0019-3124), PhD., CPsychol., AFBPsS., is Associate Professor at the University of Nottingham and Course Director for the MA in Person-Centred Experiential Counselling and Psychotherapy. David is on the British Psychological Society’s Register of Psychologists Specialising in Psychotherapy. He is editor of the international journal Person-Centered & Experiential Psychotherapies.

## Declarations

The author has declared that there are no conflicts of interest.

## Ethics Statement

This study has been approved (Reference number 2016/40/EP) by the School of Education Ethics Committee, University of Nottingham, the United Kingdom with the comment “Appropriate safeguards have been put in place for protecting the participants in this study.”

## External Funding

This article has not had any External Funding
