# Supplementary material for: Evaluation of therapeutic relationship skills training for mental health professionals: the Therapeutic Relationship Enabling Programme (TREP)
Source: MedEdPublish (2016). 2019 May 28;8:112. [Version 1] doi: 10.15694/mep.2019.000112.1 (PMC10712454; doi:10.15694/mep.2019.000112.1)
Supplement: Peer review history

# Reviewer report: Reviewer response for version 1

**DOI:** 10.21956/mep.19840.r29918

**Published:** 2021-07-14

**Authors:** Tharin Phenwan

**Affiliations:** 1 University of Dundee

This review has been migrated. The reviewer awarded 4 stars out of 5 This is an interesting innovative study aims to evaluate the training effectiveness and changes in the level of therapeutic relationship competence of the participants. The authors provided a very comprehensive and solid background in the introduction section. There are several suggestions below:Introduction-I found that the introduction section can be made more concise. The literature is relevant to this work but could be shorten. For example, at p5, the background history of Taiwan’s medical education could be shortened.Methods-Well structured and easy to follow-Several parts can be shortened. E.g. the description of sampling techniques used in this work.Results-ClearConclusions-The strengths and limitations of this study were sufficiently addressed.Structure-Several sentences can be shorten or separated into two or more sentences to ease the readers. For example, under the discussion section: ‘A further investigation on how Taiwanese mental health professional students learnt Rogerian therapeutic relationship in the intervention, in which sessions that they came to a realization of the nature of Rogerian therapeutic relationship personally and what enhanced the learning experience the most might be recommended.’‘Although there are various courses on humanity have been given in medical education, for example, seminars, group discussions and problem-based learning, Taiwanese mental health professional students’ baseline competence of therapeutic relationship still reached a degree of less satisfaction.’Having said that, I recommend this work. This will be relevant to those who are interested in experiential learning and competency and curriculum development. And I am looking forward to reading upcoming works from the authors

Reviewer Expertise:

NA

No decision status is available

# Reviewer report: Reviewer response for version 1

**DOI:** 10.21956/mep.19840.r29917

**Published:** 2019-08-22

**Authors:** Ken Masters

**Affiliations:** 1 Sultan Qaboos University

This review has been migrated. The reviewer awarded 5 stars out of 5 An exceptionally well-written and detailed evaluation of therapeutic relationship skills training intervention for mental health professionals. At times, one might feel that there is too much detail in the paper; on the other hand, it is tightly woven, and so, removing strands would threaten the flow of the paper, so it is best left as it is, even though it is a somewhat daunting read. The authors have a good and solid Introduction, laying the theoretical and educational groundwork that identifies the need for teaching aspects of psychiatry, particularly given the impact of these elements in the physician-patient relationship. This level of details sets the model for the rest of the paper, which continues in giving exceptional detail of the approach, methods, results, data analysis and then discussion. The number of participants in the workshop is a little low, and, as the authors note, the study could have benefitted from qualitative data; nevertheless, the paper itself is a meticulous and rigorous study, and deserves the highest praise.

Reviewer Expertise:

NA

No decision status is available

# Reviewer report: Reviewer response for version 1

**DOI:** 10.21956/mep.19840.r29919

**Published:** 2019-05-29

**Authors:** Felix Silwimba

**Affiliations:** 1 University of Lusaka

This review has been migrated. The reviewer awarded 5 stars out of 5 This study is applicable to types of medical fields . the findings are consistent of what is expected of trainees with regard to client -health worker interaction.

Reviewer Expertise:

NA

No decision status is available
